# Subplate Neurons as an Organizer of Mammalian Neocortical Development

**DOI:** 10.3389/fnana.2020.00008

**Published:** 2020-03-19

**Authors:** Chiaki Ohtaka-Maruyama

**Affiliations:** Neural Network Project, Department of Brain Development and Neural Regeneration, Tokyo Metropolitan Institute of Medical Science, Tokyo, Japan

**Keywords:** subplate, neocortex, development, cerebral cortex, neuronal migration

## Abstract

Subplate neurons (SpNs) are one of the earliest born and matured neurons in the developing cerebral cortex and play an important role in the early development of the neocortex. It has been known that SpNs have an essential role in thalamocortical axon (TCA) pathfinding and the establishment of the first neural circuit from the thalamus towards cortical layer IV. In addition to this function, it has recently been revealed in mouse corticogenesis that SpNs play an important role in the regulation of radial neuronal migration during the mid-embryonic stage. Moreover, accumulating studies throw light on the possible roles of SpNs in adult brain functions and also their involvement in psychiatric or other neurological disorders. As SpNs are unique to mammals, they may have contributed to the evolution of the mammalian neocortex by efficiently organizing cortical formation during the limited embryonic period of corticogenesis. By increasing our knowledge of the functions of SpNs, we will clarify how SpNs act as an organizer of mammalian neocortical formation.

## Introduction

The human brain is the most sophisticated organ in the body and carries out higher-order neural functions such as cognition, memory, and speech. The neocortex is unique to mammals and has a distinctive six-layer structure in which billions of neurons are arranged in a highly organized fashion. It is conceivable that the acquisition of this brain structure enabled the ancestral mammalian brain to evolve into the human brain, capable of highly elaborate creativity and information processing. How is this neocortex formed during embryogenesis? In order to complete the exquisite structure of the neocortex within the limited time period of embryogenesis, multiple processes, such as neurogenesis, migration, and axon targeting for early neural circuit formation, are synchronized and progress simultaneously. However, the mechanisms of this comprehensive regulation remain elusive. Subplate neurons (SpNs) are among the firstborn and matured neurons in the cerebral cortex during corticogenesis (McConnell et al., 1989; Antonini and Shatz, 1990; Ghosh et al., 1990; Kostović and Rakic, 1990; Allendoerfer and Shatz, 1994; Kanold and Luhmann, 2010; Hoerder-Suabedissen and Molnár, 2015; Duque et al., 2016). It has been reported that the number of SpNs sharply decreases in the neonatal stage in mice (Price et al., 1997; Torres-Reveron and Friedlander, 2007; Hoerder-Suabedissen et al., 2013). SpNs play a critical role in the establishment of the very first neural circuit formation between the thalamus and cortex in mid-embryonic and perinatal stages during neocortical development, suggesting their importance in the cortical formation period (McConnell et al., 1989; Ghosh et al., 1990; Kanold et al., 2003). In primates, it has been thought that SpNs remain as interstitial white matter neurons of which the cell density in human white matter shows a steep decline during the first year of life but remains relatively stable thereafter (Kostović and Rakic, 1980, 1990; Connor et al., 2009). In rodents, it has also been suggested from the morphological resemblance between SpNs and Layer 6b (L6b) neurons that SpNs survive as white matter neurons in L6b in the adult brain (Marx et al., 2017). These reports suggest that SpNs play a role not only in neocortical development but also in adult brain activity. Regarding the functions of SpNs in cortical development, it has recently been discovered that the neuronal activity of SpNs regulates the timing of the migration mode change of newborn migrating neurons (Ohtaka-Maruyama et al., 2018). Together with the known function of SpNs in thalamocortical axon (TCA) pathfinding (Ghosh et al., 1990; López-Bendito and Molnár, 2003; Hoerder-Suabedissen and Molnár, 2015), it is suggested that this cell population plays a crucial role as the metaphorical “control tower” of neocortical formation. Moreover, the subplate layer (SP layer) is abundant with extracellular matrices (ECMs) in which various signaling molecules are present (Bicknese et al., 1994; Miller et al., 1995; Maeda, 2015). This suggests that the SP layer acts as a signaling center for the formation of the cerebral cortex. The aim of this review is to summarize the known functions of SpNs to evaluate the possible role of SpNs in organizing the mammalian neocortex.

## The Earliest Born Neurons and Radial Glial Fibers Function as a Scaffold in Cortical Formation

In the same way that a scaffold is indispensable during the construction of a building and is removed upon completion, this is also, figuratively speaking, true for the brain. During neocortical formation in the embryonic stage, excitatory neurons are born from progenitors located in the ventricular zone and migrate continuously towards the pial surface, a process known as radial neuronal migration (Rakic, 1972; Tabata and Nakajima, 2003; Noctor et al., 2004; Ohtaka-Maruyama and Okado, 2015). Concurrently, mature neurons start projecting their axons to begin formation of initial neural circuits (Molnár et al., 2012). There are three cell architectures that substantially support neocortical formation: Cajal-Retzius (CR) cells, radial glial fibers, and SpNs (Luskin and Shatz, 1985; Price et al., 1997; Supèr et al., 1998). They are indispensable for neocortical formation but partly disappear due to programmed cell death after cortical formation is completed, suggesting that they function as a scaffold for mammalian brain construction. Then, how do they function to help neocortical formation? CR cells are located in the marginal zone (MZ)/layer I and secrete the extracellular protein reelin, which is involved in the regulation of radial neuronal migration (Supèr et al., 2000; Hirota et al., 2015; Frotscher et al., 2017). Radial glial fibers help the migrating neurons to move toward the pial surface (Rakic, 1972, 1988; Geschwind and Rakic, 2013). Although the role of SpNs in early cortical circuit formation, including TCA pathfinding (McConnell et al., 1989; Ghosh et al., 1990), has been reported, their other functions during the mid-embryonic stage, especially in radial neuronal migration, were still unclear.

## Known Functions of Subplate Neurons

**Figure 1:**
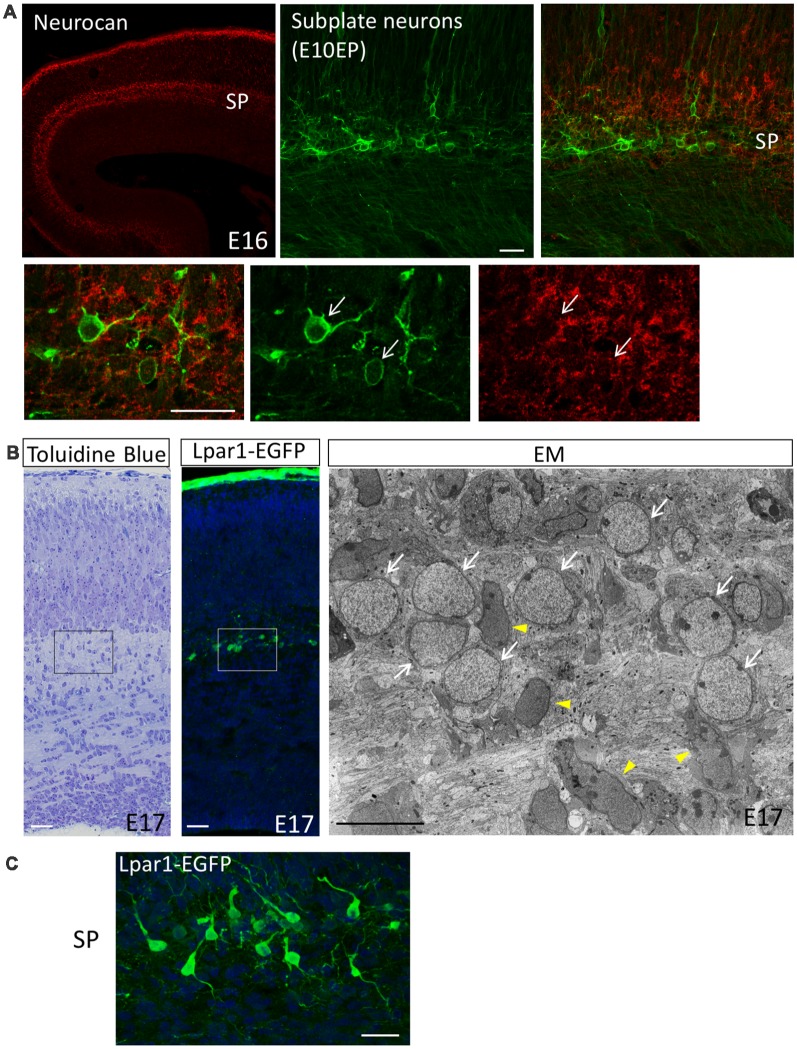
The subplate layer is rich in extracellular matrice (ECM). **(A)** Immunostaining revealed that the SP layer is rich in Neurocan (Neuronal CSPG). Subplate neurons (SpNs) were labeled with GFP (arrows) by *in utero* electroporation at E10.5 and fixed at E16. **(B)** Toluidine Blue staining, Lpar1-EGFP mouse and electron microscopic observation of the sections at E17 clearly indicate morphology of SpNs. White arrows indicate SpNs and yellow arrowheads indicate migrating neurons. **(C)** Higher magnification of SpNs of the Lpar1-EGFP mouse cortex at the E17 stage. Scale bars, 10 μm for EM image in **(B)**, 20 μm for the rest of images.

The subplate is defined as a transient zone below the cortical plate and consists of the earliest born SpNs and abundant ECMs (Figure 1; Kostović and Rakic, 1980, 1990). A well-known function of SpNs is their involvement in TCA pathfinding (TCA; Allendoerfer and Shatz, 1994; Kanold and Luhmann, 2010; Hoerder-Suabedissen and Molnár, 2015). It has been demonstrated in carnivore (McConnell et al., 1989) and rat (De Carlos and O’leary, 1992) developing brains that SpNs are the first cortical neurons to send subcortical projections across the pallial-subpallial boundary. These projections function as pioneering axons help TCAs to navigate. The ablation of SpNs by kainic acid injections or an immunotoxin to p75 prevents thalamic axons from innervating the cortex and blocks the formation of ocular dominance and orientation columns (Ghosh et al., 1990; Kanold et al., 2003). It has also been reported that the ablation of SpNs in rodents abolishes the cholinergic oscillatory activity or prevents the development of the barrel field formation of the developing cerebral cortex (Dupont et al., 2006; Tolner et al., 2012). These findings demonstrated that SpNs play a key role in neural circuit formation and cortical self-organizing processes in early developmental stages. There is a cellular diversity of SpNs in terms of their morphologies and gene expressions (Kostović and Rakic, 1980, 1990; Wahle et al., 1987; Valverde et al., 1989; Hoerder-Suabedissen and Molnár, 2013; Hoerder-Suabedissen et al., 2013). Regarding the morphological diversity, pyramidal, inverted pyramidal, multipolar, fusiform and polymorphic cells co-exist in subplate layers of mice (Figure 1C), cats and primates. Regarding axonal projection, SpNs extend their axons not only to subcortical targets but also to contralateral and ipsilateral targets inside the cortical plates (McConnell et al., 1989; De Carlos and O’leary, 1992; Mcconnell et al., 1994; Hoerder-Suabedissen and Molnár, 2012). Moreover, axonal collaterals of SpNs have a variety of arborization patterns (Friauf and Shatz, 1991; Clancy and Cauller, 1999; Myakhar et al., 2011). Regarding their molecular properties, SpNs are heterogeneous cell populations with different expression patterns of molecular markers (Hoerder-Suabedissen and Molnár, 2013; Hoerder-Suabedissen et al., 2013). This suggests that each subpopulation of SpNs plays distinct functional roles in cortical development. Moreover, it is also possible that each subpopulation is originated from distinct developmental origins. In this regard, single-cell analysis of isolated SpNs will further our understanding of their distinct characteristics. Recently, it has been reported that the layered identity of SpNs in the human fetal cortex changes post-mitotically into deep layer neurons by WNT signaling (Ozair et al., 2018). This finding may explain why the subplate layer of primates is thick in the early fetal phase but decreases in size as corticogenesis progresses, suggesting the importance of SpNs in the evolution of cortical laminar structures in higher mammals.

Despite their diversity in morphological and molecular characteristics, SpNs have relatively mature action potential characteristics. Whole-cell patch-clamp recording of SpNs in neonatal rat neocortical slices revealed resting membrane potentials and membrane resistance of −55 mV and 1 GΩ, respectively (Luhmann et al., 2000; Hirsch and Luhmann, 2008; Hanganu et al., 2009). By responding to sustained depolarizing current injection, SpNs are firing overshooting and repetitive action potentials at frequencies up to 40 Hz in rodent cortex (Hanganu et al., 2001; Unichenko et al., 2015). Using human postmortem fetal brain tissue (16–22GW), similar properties have been found that SpNs are capable of firing repetitive action potentials in response to sustained depolarization (Moore et al., 2009). Moreover, human fetal SpNs have a propensity to generate spontaneous electrical activities such as plateau depolarizations and bursts of action potential firing in spite of synaptic inputs being scarce (Moore et al., 2011). In addition to chemical synaptic inputs, the gap-junctional coupling between SpNs or SpNs and cortical neurons have been reported (Dupont et al., 2006; Singh et al., 2018). The spontaneous neuronal activity in the subplate may be derived from the depolarization of SpNs by opening connexin hemichannels in the human fetal cortex (Moore et al., 2014). These observations indicate that the spontaneous electrical events of SpNs play critical roles in the activity-dependent organization of the cerebral cortex before coherent sensory stimuli (Luhmann et al., 2009; Kanold and Luhmann, 2010; Wess et al., 2017).

## Subplate Neurons Regulate Neuronal Migration

We have recently discovered that SpNs are also involved in the regulation of the radial neuronal migration process in mice (Ohtaka-Maruyama et al., 2018). One conventional method to examine the function of a gene is by introducing si-RNA or knockdown constructs into neural progenitor cells located in the ventricular zone using *in utero* electroporation (Tabata and Nakajima, 2001). Using this technique, we have found that knockout of the gene encoding for the transcriptional repressor RP58 resulted in impairment of radial migration and that the knockout neurons stopped migrating in the middle of the cortex (Ohtaka-Maruyama et al., 2013). Subsequently, we found that knockout or knockdown of many other different genes such as small G-protein regulators, microtubule-binding proteins or the modifying enzyme of the sugar chain, exhibit a similar phenotype (Ohtaka-Maruyama and Okado, 2015). Those migration-defective neurons often stop migrating in the middle of the cortex and remain just under a certain boundary. This led to the question “what is this boundary?” Various antibodies were used to immuno-stain different cortical layers, and MAP2 immunostaining revealed that the boundary is the SP layer. However, it is still not known why migration-defective neurons stop just under the SP.

We observed the behavior of migrating neurons labeled by *in utero* electroporation using time-lapse imaging of cultured cortical slices and found that migrating multipolar neurons of wild types also pause just under the SP after multipolar migration, followed by a shift into the locomotion mode. This suggests that the SP layer is the location for the conversion of migrating neurons from multipolar to bipolar as well as the change in their migration mode. The SP layer consists of SpNs and abundant ECMs (Hoerder-Suabedissen et al., 2013; Maeda, 2015). We hypothesized that SpNs send signals to neurons in multipolar migration mode to convert their morphology into bipolar shape and switch their migration mode into locomotion.

## Sp Neurons Interact With Migrating Neurons *via* Transient Synapse-Like Contacts

**Figure 2:**
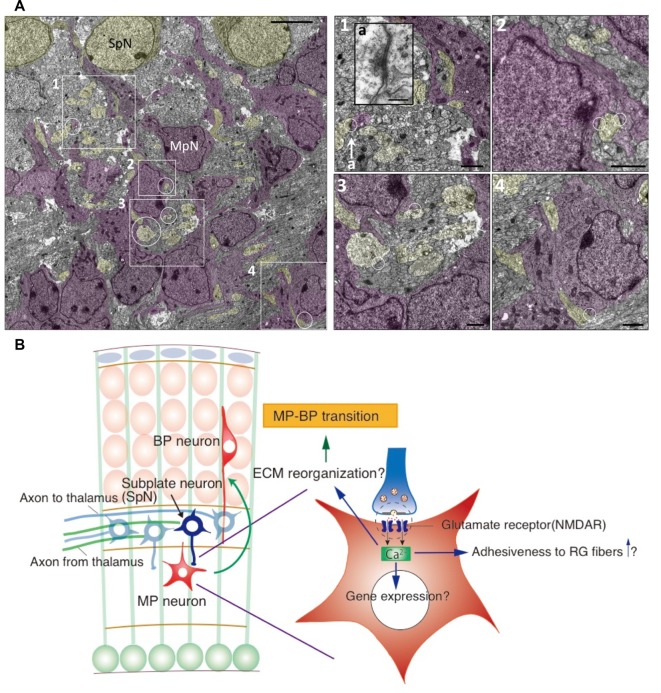
Synaptic interaction between SpNs and MpNs is found just below the subplate layer. **(A)** Electron microscopic images of synapses on MpNs in the E16 cortical section. The areas indicated by the rectangles and circles are enlarged in the right. Synapse located in 1-a is enlarged as an inset. Scale bars, 50 μm for the left image, 200 nm for a, 10 μm for 1–4. **(B)** Hypothetical model for the interaction between SpNs and multipolar neurons. SpNs form transient glutamatergic synapses on multipolar neurons, which induce a Ca^2+^ influx in multipolar neurons through the NMDA receptor. Calcium signaling may alter gene expressions involved in cytoskeletal remodeling and cell adhesiveness, leading to multipolar-to-bipolar transition (Ohtaka-Maruyama et al., 2018).

We first examined the possibility that Ca^2+^ signaling is involved in the conversion of the migration mode. A GCAMP3 construct was introduced into migrating neurons and time-lapse imaging of the cultured slices was performed. The calcium concentration of migrating neurons was transiently increased when they passed through the SP layer, followed by the conversion of their migration mode into the locomotion mode. This suggests that newborn neurons migrating in the multipolar migration mode receive signals at the SP layer and that their intracellular concentration of Ca^2+^ is increased. We hypothesized that SpNs are sending signals to migrating neurons causing the intracellular concentration of Ca^2+^ to transiently increase. We then examined if SpNs are neuronally active during the mid-embryonic stage using GCaMP plasmids, which were introduced into SpNs by *in utero* electroporation at E10.5. Time-lapse imaging of cultured slices prepared from electroporated mice revealed that SpNs are already neuronally active at E15, as evidenced by Ca^2+^ oscillations. We also examined and confirmed that SpNs release presynaptic vesicles using a synapto-pHluorin probe. Subsequently, the interaction between SpNs and migrating neurons was examined by time-lapse imaging and electron microscopy. Surprisingly, it was revealed that a synapse-like adhesive structure is formed between neurite of SpN (presynaptic-like) and cell body of migrating neuron (postsynaptic-like; Ohtaka-Maruyama et al., 2018; Figure 2A). Many synaptic boutons are formed by TCAs around SpN cell bodies (Herrmann et al., 1994). We could distinguish these synapses from the synaptic junctions formed on MpNs by electron microscopic images as the synapses around SpNs were characterized by more synaptic vesicles and pale postsynaptic cytoplasm with fewer ribosomes compared with those around MpNs (Ohtaka-Maruyama et al., 2018). Moreover, we confirmed that these synapses on MpNs are formed by presynaptic contacts from SpNs by performing double-label immunoelectron microscopy (Ohtaka-Maruyama et al., 2018). The next question was, “Is this synapse-like contact involved in the regulation of radial neuronal migration?” To answer this question, we suppressed the activity of SpNs to see the effect on radial migration and found that radial migration, especially into the CP, was impaired. This suggests that the neuronal activity of SpNs is critical for changing the migration mode. In support of this, knockdown of the post-synaptic proteins NR1 and PSD-95 also affected radial migration. Finally, we examined the effect of local application of glutamate on multipolar cells. Glutamate uncaging reagent was used to release glutamate locally in cultured slices prepared from mouse brains electroporated with Kik-GR plasmid. The switch to the locomotion mode was facilitated in multipolar cells with locally applied glutamate compared with controls. Based on these findings, we propose the following model. Newborn neurons migrating toward the SP layer receive signals from neurites of SpNs *via* transient synapse-like interactions. This induces Ca^2+^-influx into newborn neurons that drive intracellular signaling producing cytoskeletal remodeling. This leads to a transition to a bipolar shape and increases the adhesiveness to RG fibers, promoting switching to the locomotion mode (Ohtaka-Maruyama et al., 2018; Figure 2B). Synapses transmit electric signals among mature neurons. However, our results demonstrate that synaptic transmission occurs in the fetal brain and induces morphological and movement mode changes in young neurons in the mouse developing cortex.

## Where do Subplate Neurons Originate?

SpNs are generated between E10.5 and E12.5 in mice (Price et al., 1997; Hoerder-Suabedissen and Molnár, 2013). Initially, it was thought that SpNs are derived from ventricular zone progenitors. However, it has since been revealed that SpNs are also generated from Tbr2-positive intermediate progenitors located in the subventricular zone (Vasistha et al., 2015). It has also been reported that a cohort of SpNs is migrating from the rostromedial telencephalic wall (Pedraza et al., 2014). These findings support the idea that SpNs contain heterogeneous subpopulations. Indeed, subpopulations of SpNs exhibit different expression patterns and that there is an overlapping pattern of four well-defined SpN markers, Complexin3, CTGF, Nurr1, and Lpar1 (Hoerder-Suabedissen et al., 2009; Hoerder-Suabedissen and Molnár, 2013). Subpopulations of SpNs expressing specific molecular markers exhibit specific projection patterns (Grant et al., 2012; Viswanathan et al., 2017; Hoerder-Suabedissen et al., 2018). Moreover, GABAergic interneurons also reside in the SP layer and are born in the medial ganglionic eminence and migrate into the SP layer by tangential migration. This suggests that the heterogeneity of SpNs supports their multiple functions and that they are derived from various evolutionary origins (Montiel et al., 2011).

## Subplate Neurons Function as an Organizer of Neocortex Development

As described above, SpNs play multiple roles in a cortical formation. For example, Lpar1-EGFP-positive SpNs extend their neurites towards migrating neurons, but rarely extend subcortical projections during this embryonic stage (Grant et al., 2012; Ohtaka-Maruyama et al., 2018). This subpopulation participates in the regulation of radial migration. In contrast, in another subplate-specific transgenic mouse line, Golli-tau-eGFP-positive neurons project corticofugal fibers from the early embryonic stage at E13.5, suggesting that this subpopulation is involved in the establishment of thalamocortical connections (Jacobs et al., 2007; Grant et al., 2012). It has been known that a certain population of SpNs survive until adulthood. L6b neurons (white matter neurons), which are considered to be the remnants of SpNs, innervate higher-order thalamic nuclei in mice (Hoerder-Suabedissen et al., 2018). Moreover, L6b neurons are the only neuronal population responsive to orexin (hypocretin), a key molecule in regulating the sleep-wake cycle (Lin et al., 1999; Bayer et al., 2004). These findings suggest that SpNs are not just remnants, but play a role in the modulation of neural circuits in the adult brain. Taken together, these studies indicate that SpNs act as an organizer of cortical development by orchestrating neocortical formation, cell migration, and the initial wiring and modulation of neural circuits during cerebral cortical development.

## Evolution of the Cerebral Cortex and the Subplate

Although the SP layer is unique to mammals, the expression of molecular markers for SpNs is observed in sauropsid brains (Montiel et al., 2011; Wang et al., 2011). This suggests a subpopulation of SpNs in the ancestral amniote cortex (Marín-Padilla, 1971; Montiel et al., 2011), which evolved in the mammalian cortex by adding new populations that exhibit a layer structure. In this context, the layer structure of the mammalian subplate may have been important for the evolution of an elaborate neocortex containing more complex wiring. Comparison of the thalamic projections toward the dorsal cortex between turtles and evolutionary primitive mammals (opossums, hedgehogs) revealed greater thalamic projections to the dorsal cortex than the SP layer compared with the MZ during evolution (Hall and Ebner, 1970; Supèr et al., 1998). As there is no subplate as a layer in the turtle cortex, this finding suggests that the SP layer played an important role in TCA guidance during the evolution of the mammalian neocortex.

The SP layer is much thicker in primates than in mice (Kostović and Rakic, 1990). This suggests that the SP layer has more functions in higher-order animal brains. In human brain development, histological and MRI studies of post-mortem brain samples or MRI images obtained from either *in utero* scanned fetuses or prematurely born infants revealed that the SP layer expands from 13 to17 PCW, to become the most prominent layer in the telencephalon (the peak of SP development is between 26–31 PCW; Kostović et al., 2002, 2014; Bayatti et al., 2008). After 35 PCW, the volume of the SP layer decreases and a distinct SP layer cannot be identified by around 6 months after birth, although remnants of SpNs are thought to remain as interstitial white matter cells (Kostović and Rakic, 1990; Hoerder-Suabedissen and Molnár, 2015; Kostović et al., 2018). Together with the finding that there are subpopulations of SpNs in mice, additional novel subpopulations of SpNs may have developed in higher-order mammals and primates with gyrencephalic brains. These novel cohorts of SpNs may have contributed to the elaborate wiring of gyrification. Further investigation, such as identification of primate-specific cell populations or genetic markers for SpNs, would help to reveal the role of SpNs in cortical organization and their contribution to evolution in higher-order mammals and humans. Moreover, it will clarify the functional differences of subpopulations of SpNs, increasing our understanding of the importance of SpNs and revealing the precise mechanisms of neocortical development and evolution.

## Subplate Function and Brain Developmental Disorders

As described above, the human fetal SpNs are spontaneously active at midgestation which is earlier than sensory input from the thalamus has reached the cortex (Moore et al., 2011). These early activities before birth are thought to be critical for fundamental processes of brain development including neurogenesis, neuronal migration, axon pathfinding, dendrite patterning, transmitter selection and ion channel development (Moody and Bosma, 2005; Spitzer, 2006). In rodents, SpNs promote spindle bursts and thalamocortical patterning in the neonatal rat somatosensory cortex (Dupont et al., 2006; Tolner et al., 2012). These findings again suggest that SpNs act as an organizer to influence the following developmental events during cortical formation in human cortical development. Fetuses and newborn babies exhibit spontaneous involuntary body movement called “General movement” that is likely derived from spontaneous activity in the developing neocortex (Prechtl, 2001). The voluntary movement gradually supersedes general movement after 3 months of age, which corresponds with the decrease of the SP layer, suggesting that general movement is a reflection of spontaneous activity in early-born neurons including SpNs (Bayatti et al., 2008; Hadders-Algra, 2018). Many studies have reported that observation of general movement could be useful to predict developmental delay or autism that may occur at later ages (Zuk, 2011; Kanemaru et al., 2013; So et al., 2016; Gima et al., 2018). Moreover, the finding that genes expressed in a subplate-specific manner in mice are significantly associated with autism spectrum disorders and schizophrenia (Hoerder-Suabedissen et al., 2013) supports the notion that general movement in fetuses and neonatal human babies is a reflection of the spontaneous activity of SpNs.

## Concluding Remarks

Recent advances have revealed crucial roles for SpNs in brain formation and function during cortical development, including in neuronal migration and axon wiring involved in sleep-wake cycles. Future clarification of SpN subpopulations at the single-cell level may identify subplate-specific molecular markers at early stages. This would make it possible to establish better SpN-specific Cre mouse lines and to explore the developmental and evolutionary origins of subplate cells in more detail. Moreover, future cooperative projects incorporating basic research using experimental animals and clinical research during child development will advance our understanding of how SpN activity affects the development of neurodevelopmental and psychiatric disorders during corticogenesis.

## Author Contributions

CO-M wrote the manuscript.

## Conflict of Interest

The author declares that the research was conducted in the absence of any commercial or financial relationships that could be construed as a potential conflict of interest.
